# Prescribing trends and patterns for antihypertensive agents in primary healthcare settings in Qatar: a retrospective observational study

**DOI:** 10.1080/20523211.2025.2512183

**Published:** 2025-06-18

**Authors:** Nada Nabil Abdelkader, Ahmed Awaisu, Hazem Elewa, Ziyad Mahfoud, Samya Ahmad Al Abdulla, Amjad Owais, Maguy Saffouh El Hajj

**Affiliations:** a Clinical Pharmacy and Practice Department, College of Pharmacy, QU Health, Qatar University, Doha, Qatar; b Biostatistics and Healthcare Policy and Research, Weill Cornell Medicine, Qatar Foundation, Doha, Qatar; c Primary Health Care Corporation, Doha, Qatar

**Keywords:** Hypertension, prescribing patterns, prescribing trends, antihypertensives, Qatar

## Abstract

**Background:**

An observational retrospective study was conducted in the primary care setting in Qatar to evaluate the prescribing trends and patterns of antihypertensives in hypertensive patients.

**Methods:**

Included patients were adult patients (aged ≥18 years) diagnosed with essential hypertension and attending Primary Health Care Corporation (PHCC) clinics in Qatar between January 2017 and March 2021. A sample of 2185 patients was randomly selected. The data were collected from PHCC’s electronic medical records system, Cerner©.

**Results:**

Angiotensin Converting Enzyme inhibitors (ACEIs) prescriptions decreased from 38% in 2017 to 21% in 2021, and that of Angiotensin Receptor Blockers (ARBs) from 37% in 2017 to 31% in 2021. Patients with prescribed Calcium Channel Blockers (CCBs) increased from 31% in 2017 to 40% in 2021. The prescribing of thiazide diuretics declined from 32% in 2017 to 9% in 2021. In 2017, the most prescribed antihypertensive class was ACEIs (38%), while the least prescribed antihypertensive class was loop diuretics (1.4%). In 2018, the most prescribed class of antihypertensives was CCBs (36%) and the least prescribed was vasodilators (0.1%). In 2019, 2020 and 2021, CCBs were the most prescribed.

**Conclusion:**

These findings are consistent with national and international guidelines for hypertension management and with published literature. Yet, there is still room for improvement to optimise antihypertensive prescribing practices. Prescribers need to address potential gaps and explore ways to enhance their hypertension management, so that patients have access to effective and evidence-based treatments.

## Background

According to the World Health Organisation (WHO) report data in 2023, around 1.28 billion adults aged 30–79 years were reported to have hypertension, with about two-thirds residing in low and middle-income countries (LMICs) (Hypertension key facts, 2021). Hypertension is a preventable risk factor for morbidity and mortality, and when not diagnosed early and treated appropriately, it may lead to fatal complications (Ambrose & Barua, 2004; Maclaughlin). Hypertension ranked sixth among the leading causes of death and disability in Qatar in 2018 (World Health Organization. Noncommunicable diseases country profiles, 2018). In 2019, another study using the Qatar Biobank (QBB) database revealed that dyslipidemia (30.1%), diabetes (17.4%), hypertension (16.8%), and asthma (9.1%) were the most prevalent Noncommunicable diseases (NCDs) in Qatar (Al Thani et al., 2019). According to a review published in 2021, the prevalence of hypertension among adults in Qatar was found to reach 32% (16.8%−32.1%) (AbdulRashid et al., 2021).

Appropriate control of blood pressure (BP) is imperative to avoid potentially fatal complications associated with hypertension. BP lowering agents (i.e. antihypertensive medications) can significantly lower the risk of cardiovascular-related morbidity and premature deaths.(Ettehad et al., 2016). A meta-analysis of randomised clinical trials (RCTs) evaluating antihypertensives effects on BP over time and among varying participant characteristics, revealed that antihypertensives were effective in BP lowering at a maximal effect 12 months post-initiation and the effect was further reduced gradually for years later (Canoy et al., 2022). Several national and international clinical practice guidelines (CPGs) have been published to provide guidance to clinicians on hypertension management worldwide. Examples of these guidelines include: the Eighth Joint National Committee Guideline (JNC8), the National Institute for Health and Care Excellence Hypertension in Adults Guideline (NICE), the American College of Cardiology/American Heart Association ACC/AHA, and the European Society of Cardiology/ European Society of Hypertension guideline (ESC/ESH) (Cuspidi et al., 2018; Dorans et al., 2018; James et al., 2014; National Institute for Health and Care, 2019). Different CPGs may have varying and diverse recommendations for hypertension management.

In the State of Qatar, the health care system comprises the governmental and the private sectors. The governmental sector is mainly composed of Hamad Medical Corporation (HMC), which includes tertiary and secondary care hospitals, as well as outpatient primary health care corporation (PHCC) centres (Health Care in Qatar, 2023). The majority of ambulatory hypertensive patients in the country are treated at PHCC centres. In 2016, the first guidelines for the management of hypertension were developed by the Qatar Ministry of Public Health (MOPH) in collaboration with HMC and practitioners from both private and other governmental sectors (Clinical Guidelines for the State of Qatar, 2019). The PHCC developed its own hypertension management guidelines while using the JNC8, and NICE guidelines as the basis for its recommendations. The PHCC guidelines are revised every three years or whenever new international hypertension guidelines emerge. The latest guidelines for the management of hypertension in adults at PHCC were last revised in 2020 and the next revision is expected to be published in late 2023 (Primary Health Care Corporation, 2020). These updated PHCC guidelines are based on both the NICE guidelines and the MOPH guidelines (Clinical Guidelines for the State of Qatar, 2019; National Institute for Health and Care Excellence, 2019). According to these guidelines, patients with specific underlying conditions may benefit from tailored antihypertensive therapy. For example, patients with Chronic Kidney Disease (CKD) and albuminuria are recommended to initiate treatment with ACEIs or Angiotensin Receptor Blockers (ARBs), either as a primary option or in combination therapy. These guidelines also advocate for blood pressure (BP) targets below 140/90 mmHg for patients under 80 years of age, while for those aged 80 and older, the recommended BP target is 150/90 mmHg (Primary Health Care Corporation, 2020).

Despite the availability of hypertension management guidelines in Qatar, there is a lack of clear information related to how hypertension is managed, especially in the context of primary care. Furthermore, to our knowledge there are no studies or national morbidity surveys or registries regarding the prescribing trends and patterns of antihypertensive agents in general hypertensive patients or patients with comorbidities in the country. Therefore, the objectives of this study were to evaluate antihypertensives’ prescribing trends and patterns in general hypertensive patients and hypertensive patients with comorbidities in the primary care setting in Qatar.

## Methods

### Study setting

The study was conducted at primary health centres under the umbrella of PHCC in Qatar. The PHCC operates 31 primary healthcare centres throughout the State of Qatar. At the time of the study, there were 13 centres in Doha, the capital city of Qatar, and the remaining 18 centres were distributed throughout the country. These centres are the main providers of healthcare for patients with Non-Communicable Diseases (NCDs), including hypertension, diabetes, and dyslipidemia (Primary Health Care Corporation, n.d.).

### Study design

This study was a retrospective observational study that was conducted in all PHCC centres in Qatar. A retrospective study design was proposed as using a prospective design would necessitate a long study duration and manpower to achieve the study’s objectives.

### Study participants

The inclusion criteria for the study included adult patients (aged ≥18 years) who were diagnosed with essential hypertension between January 2017 and March 2021. Patients were excluded if antihypertensives were prescribed for reasons other than hypertension, or if they had secondary or pregnancy-induced hypertension. The data for the study were obtained from Cerner© electronic health record (EHR) software used in the study setting. Through this database, the medical records of included patients were identified using the International Classification of Diseases (ICD) 11 coding for essential hypertension (*ICD-11*, n.d.).

### Sample size determination

The study aimed to evaluate antihypertensives prescription patterns and trends at PHCC centres in Qatar. With an estimated 2000 patients, 500 per year, the study was able to estimate the yearly prevalence of antihypertensive prescriptions within a margin of error of at most 4.5% using 95% confidence intervals (CI). In addition, with this sample size, the study will be able to detect an increasing or decreasing trend of at least 2% per year using the Cochrane Armitage trend test with a power of 80% and an alpha level of 5%.

### Sampling technique

A simple random sampling technique was utilised to select adult patients with essential hypertension attending PHCC centres through Cerner©.

### Data collection procedure

The information technology (IT) team at the PHCC identified patients with essential hypertension through Cerner© and randomly selected a sample of 2185 patients. The IT team provided the research team with an Excel© sheet containing all included patients’ sociodemographic, medication, and medical information based on a predesigned data collection form. Patients were anonymised to remove identifying information such as names, health card numbers, or contact information. The medical information of included patients encompassed their medication lists, comorbidities, and prescriptions. Antihypertensive prescriptions were specifically extracted and systematically recorded in an Excel© sheet for each year. This approach facilitated the analysis of prescribing patterns and trends for antihypertensive medications among hypertensive patients at PHCC centres in Qatar.

The study team reviewed the data and contacted the IT team if more information was required to ensure proper and comprehensive data collection. Frequent monitoring was conducted by the research team to ensure data collection was done as per the study protocol through weekly meetings.

### Data analysis

Patients’ demographic and clinical characteristics were summarised using mean and standard deviation with minimum and maximum values for age and frequency distribution for all categorical variable (e.g.: gender, country of origin, comorbidities and date of diagnosis). Frequency distributions were used to summarise the percentage of patients with specific medications over time. This was also stratified for subgroups of patients with specific conditions. Overall, trends over time were tested for specific medications using the Cochran–Armitage test for trend. Statistical significance was set at the 5% level. All analyses were done using IBM-SPSS (version 28, Armonk, NY, USA) and STATA (version 17, College Station, TX, USA). Patterns and trends were analyzed out of the number of hypertensive patients receiving treatment (‘N’). ‘N’ under each year does not align with the number of patients diagnosed with hypertension in that specific year because some hypertensive patients may not have been started on therapy immediately after diagnosis and may have initially used lifestyle modifications.

## Results

### Characteristics of patients with hypertension attending primary care setting in Qatar

Two thousand one hundred eighty-five medical charts were randomly selected from patients diagnosed with essential hypertension in 2017, 2018, 2019, and 2020: 547, 544, 545, and 549, respectively. The mean (SD) age of the study sample was 57.91 (12.32) years. About half of the patients (49.3%) were Qatari nationals, while the rest were from diverse nationalities. The most common comorbidity among the study subjects was diabetes mellitus (65.4%) followed by CVD (57.9%) (Table 1).

**Table 1.** Sociodemographic characteristics of patients.

Characteristic	Frequency (Per cent)
**Age (*N* = 2185)** Mean (SD) Range: Minimum-Maximum	57.91 (12.32) 19.0-97.0
**Gender (*N* = 2185)** Male Female	1025 (46.9%) 1160 (53.1%)
**Country of origin (*N* = 2185)** Qatar India Egypt Pakistan Philippines Sudan Jordan Palestine Bangladesh Syria Other countries^a^	1077 (49.3%) 199 (9.1%) 148 (6.8%) 117 (5.4%) 83 (3.8%) 78 (3.6%) 73 (3.3%) 70 (3.2%) 66 (3.0%) 39 (1.8%) 235 (10.7%)
**Comorbidities^b^(*N* = 2185)** Diabetes Dyslipidemia CKD CHF CVDs AFib Other Arrhythmias PVD **Attending Smoking Clinic (Yes)**	1428 (65.4%) 1265 (57.9%) 218 (10.0%) 113 (5.2%) 106 (4.9%) 82 (3.8%) 30 (1.4%) 21 (1.0%) 24 (1.1%)
**Date of Hypertension Diagnosis (*N* = 2185)** 2017 2018 2019 2020	547 (25.0%) 544 (24.9%) 545 (24.9%) 549 (25.1%)

^a^ Afghanistan, Algeria, Australia, Bahrain, Canada, Eritrea, Ethiopia, France, Ghana, Indonesia, Iran, Iraq, Italy, Kenya, Kuwait, Lebanon, Malaysia, Mexico, Morocco, Nepal, Nigeria, Oman, Saudi Arabia, Somalia, South Africa, Sri Lanka, Sweden, Tunisia, Turkey, United Arab Emirates, United Kingdom, United States of America, Yemen.

^b^ IHDs: Ischemic Heart Diseases CHF: Congestive Heart Failure; CKD: Chronic Kidney Disease; PVD: Peripheral Vascular Disease; Afib: Atrial Fibrillation.

### Antihypertensives prescribing trends in primary health care setting in Qatar

**Figure 1.**
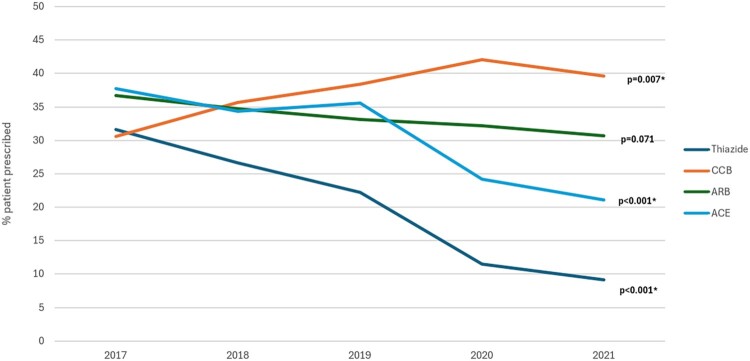
Prescribing trends of antihypertensives among patients attending primary care setting in Qatar. ACEIs: angiotensin-converting enzyme inhibitors; CCBs: calcium channel blockers; ARBs: angiotensin receptor blockers.

Antihypertensive prescribing trends from 2017 to 2021 for specific hypertensive classes at PHCC in Qatar are found in Figure 1. There were significant decreases in ACEIs and Thiazide diuretics from the year 2017 to 2021 (*p*-values were both <0.001). In particular, ACEIs were prescribed to 37.8% of the patients in 2017, and this declined to 21.1% in 2021. Similarly, thiazides were prescribed to 31.6% of patients in 2017 and decreased to 9.1% in 2021. On the other hand, there was a significant increase (*p* = 0.007) in prescribing CCBs to patients from 30.6% in 2017 to 39.6% in 2021. The percentage of ARBs prescribed patients decreased from 36.7% in 2017 to 30.7% in 2021, but such a decrease didn’t reach statistical significance (*p* = 0.071) (See Figure 1 for more details).

### Overall antihypertensives prescribing patterns in primary care setting in Qatar

The overall prescribing pattern for different antihypertensive classes was examined from 2017 to 2021. In 2017, ACEIs were the most commonly prescribed antihypertensive class (26.6%), whereas loop diuretics were the least prescribed (1.4%). However, in 2018, the most commonly prescribed class of antihypertensives was CCBs (22.9%) and the least prescribed was vasodilators (0.1%). This pattern remained similar in 2019, where CCBs accounted for 26.5% of all antihypertensive prescriptions. In 2020 and 2021, CCBs were the most commonly prescribed antihypertensive agents, accounting for nearly one-third of prescriptions, while vasodilators were the least prescribed (0.3% of all prescriptions).

#### Overall antihypertensives prescribing pattern regardless of comorbidities

In 2017, 98 patients diagnosed with hypertension received drug therapy. Of this, 53 patients (54.1%) received monotherapy, while 32 patients (32.6%) received dual therapy. ARBs were the most commonly prescribed antihypertensives among those treated with monotherapy (35.8%), followed by ACEIs (28.3%). As for dual therapy combinations, the most common combinations were ACEIs + Thiazides (25.0%) and ACEIs + CCBs (21.9%).

In 2018, 731 patients with hypertension received drug therapy. Of these, 418 patients (57.2%) received monotherapy, whereas 224 patients (30.6%) received dual therapy. Among those on monotherapy, ACEIs (28.9%) and CCBs (25.4%) were the most frequently prescribed antihypertensives. On the other hand, the most commonly prescribed dual therapy was ARBs + Thiazides (25.0%), followed by ACEIs + CCBs (18.8%).

In 2019, there were 959 patients treated for hypertension, of which 599 (62.5%) were on monotherapy and 269 (28.0%) were on dual therapy. Among monotherapy patients, ACEIs (29.7%) and CCBs (28.4%) were the most common antihypertensives prescribed. Among patients receiving dual therapy, the most prescribed combination was ACEIs + CCBs (23.5%), followed by ARBs + Thiazides (22.8%).

Further details on antihypertensives patterns in the general population are presented in Table 2.

**Table 2.** Overall patterns of antihypertensive therapy in patients with hypertension attending primary care in Qatar (*N* = 2185).

Number of treated hypertensive patients					
	2017 *N* = 98	2018 *N* = 731	2019 *N* = 959	2020 *N* = 1144	2021* N = 560
**Mean SBP^a^(SD)**	**	**134.2 (14.5)**	**133.0 (13.7)**	**134.3 (15.7)**	**–**
**Mean DBP^b^(SD)**	**	**76.3 (8.3)**	**75.3 (8.6)**	**76.3 (9.4)**	**–**
**Monotherapy^c^*n* (%)**	**53(54.1%)**	**418(57.2%)**	**599(62.5%)**	**864(75.5%)**	**471(84.1%)**
BB	7 (13.2%)	47 (11.2%)	54 (9.0%)	58 (6.7%)	36 (7.6%)
ACEI	15 (28.3%)	121 (28.9%)	178 (29.7%)	199 (23.0%)	102 (21.7%)
CCBs	8 (15.1%)	106 (25.4%)	170 (28.4%)	301 (34.8%)	163 (34.6%)
Loop diuretics	1 (1.9%)	22 (5.3%)	21 (3.5%)	33 (3.8%)	16 (3.4%)
Thiazides	3 (5.7%)	19 (4.5%)	24 (4.0%)	38 (4.4%)	22 (4.7%)
ARBs	19 (35.8%)	102 (24.4%)	149 (24.9%)	229 (26.5%)	125 (26.5%)
Alpha2 agonists	–	1 (0.2%)	4 (0.7%)	6 (0.7%)	4 (0.8%)
Imidazoline receptor agonists	–	1 (0.2%)	–	2 (0.2%)	1 (0.2%)
Direct vasodilators	–	–	–	1 (0.1%)	1 (0.2%)
**Dual Therapy^c^*n* (%)**	**32(32.6%)**	**224(30.6%)**	**269(28.0%)**	**216(18.9%)**	**74(13.2%)**
CCB + ACEI	7 (21.9%)	42 (18.8%)	63 (23.5%)	35 (16.2%)	6 (8.1%)
ARBs + Thiazides	6 (18.8%)	56 (25.0%)	61 (22.8%)	28 (12.9%)	9 (12.2%)
BBs + Thiazides	2 (6.3%)	6 (2.7%)	5 (1.8%)	2 (0.9%)	2 (2.7%)
ACEIs + Thiazides	8 (25.0%)	38 (17.0%)	45 (16.8%)	7 (3.2%)	1 (1.4%)
ACEIs + BBs	4 (12.5%)	11 (4.9%)	9 (3.3%)	12 (5.6%)	2 (2.7%)
CCBs + ARBs	2 (6.3%)	22 (9.8%)	31 (11.5%)	50 (23.2%)	21 (28.4%)
ACEIs + ARBs	–	3 (1.3%)	4 (1.5%)	–	–
ACEIs + Loop diuretics	–	1 (0.5%)	5 (1.8%)	6 (2.8%)	2 (2.7%)
BBs + ARBs	1 (3.1%)	9 (4.0%)	10 (3.7%)	10 (4.6%)	4 (5.4%)
BBs + CCBs	1 (3.1%)	12 (5.4%)	10 (3.7%)	15 (6.9%)	5 (6.8%)
BBs + Loop diuretics	–	4 (1.8%)	5 (1.8%)	6 (2.8%)	1 (1.4%)
Loop diuretics + ARBs	1 (3.1%)	5 (2.2%)	7 (2.6%)	8 (3.7%)	3 (4.0%)
Thiazides + CCBs	–	10 (4.5%)	12 (4.5%)	18 (8.3%)	9 (12.2%)
Loop diuretics + CCBs	–	2 (0.9%)	2 (0.7%)	10 (4.6%)	6 (8.1%)
Other dual therapy combinations	–	3 (1.3%)	–	9 (4.2%)	3 (4.1%)
**Triple therapy*n* (%)**	**10(10.2%)**	**73(9.9%)**	**73(7.6%)**	**56(4.9%)**	**12(2.1%)**
**Quadruple therapy *n* (%)**	**3(3.0%)**	**13(1.8%)**	**14(1.5%)**	**8(0.7%)**	**3(0.5%)**

*BP data was collected up till 30th of March 2021.

**Missing readings.

^a^ SBP: Systolic BP.

^b^ DBP: Diastolic BP.

^c^ BBs: Beta-blockers; ACEIs: Angiotensin-converting enzyme inhibitors; CCBs: Calcium channel blockers; ARBs: Angiotensin receptor blockers; Vasodilators: Hydralazine; Imidazoline receptor agonists: Moxonidine; Multiple drug therapy refers to patients receiving more than 4 antihypertensives drug therapy.

#### Antihypertensives prescribing patterns among patients with hypertension and diabetes

Antihypertensives were prescribed to 82 patients with hypertension and diabetes in 2017. Forty-three patients (52.4%) received monotherapy and 27 patients (32.9%) received dual therapy. ACEIs and ARBs were equally prescribed among the population treated with monotherapy (34.9% each). It was found that ACEIs + thiazides (29.6%) and ACEIs + CCBs (22.2%) were the two most common dual therapy combinations.

There were 538 patients with hypertension and diabetes who received antihypertensives in 2018 with 299 patients (55.6%) on monotherapy and 167 patients (31.0%) on dual therapy. The most commonly prescribed antihypertensives among patients on monotherapy were ACEIs (34.8%), followed by ARBs (26.8%). In patients on dual therapy, the most common combination was ARBs + Thiazides (28.7%), followed by ACEIs + CCBs (18.0%).

In 2019, there were 633 patients with hypertension and diabetes who received treatment. Of these, 390 (61.6%) were on monotherapy, and 174 (27.5%) on dual therapy. Of those receiving monotherapy, ACEIs (34.4%) were the most commonly prescribed antihypertensives, followed by ARBs (27.9%). Among the most prescribed dual combinations, ARBs + Thiazides accounted for 24.7%, followed by ACEIs + CCBs (16.1%).

Further details on antihypertensives patterns in patients with diabetes comorbidity are illustrated in Table 3.

**Table 3.** Patterns of antihypertensive in patients with diabetes and hypertension (*N* = 1428).

	Number of treated hypertensive patients				
	2017 *N* = 82	2018 *N* = 538	2019 *N* = 633	2020 *N* = 728	2021* N = 376
**Mean SBP**^a^**(SD)**	**	**134.2 (14.5)**	**133.0 (13.7)**	**134.3 (15.7)**	**–**
**Mean DBP**^b^**(SD)**	**	**76.3 (8.3)**	**75.3 (8.6)**	**76.3 (9.4)**	**–**
**Monotherapy**^c^***n* (%)**	**43** (**52.4%)**	**299** (**55.6%)**	**390** (**61.6%)**	**529** (**72.6%)**	**306** (**81.4%)**
BB	5 (11.6%)	29 (9.7%)	38 (9.7%)	38 (7.2%)	27 (8.8%)
ACEI	15 (34.9%)	104 (34.8%)	134 (34.4%)	151 (28.5%)	79 (25.8%)
CCBs	5 (11.6%)	56 (18.7%)	78 (20.0%)	142 (26.8%)	80 (26.1%)
Loop diuretics	–	21 (7.0%)	19 (4.9%)	29 (5.5%)	15 (4.9%)
Thiazides	3 (7.0%)	10 (3.3%)	11 (2.8%)	19 (3.6%)	12 (3.9%)
ARBs	15 (34.9%)	80 (26.8%)	109 (27.9%)	149 (28.2%)	89 (29.1%)
Alpha2 agonists	–	–	1 (0.3%)	1 (0.2%)	1 (0.3%)
Imidazoline receptor agonists	–	–	–	2 (0.4%)	1 (0.3%)
Direct vasodilators	–	–	–	1 (0.2%)	1 (0.3%)
**Dual Therapy**^c^ ***n* (%)**	**27** (**32.9%)**	**167** (**31.0%)**	**174** (**27.5%)**	**150** (**20.6%)**	**58** (**15.4%)**
CCB + ACEI	6 (22.2%)	30 (18.0%)	28 (16.1%)	17 (11.3%)	3 (5.2%)
ARBs + Thiazides	4 (14.8%)	48 (28.7%)	43 (24.7%)	19 (12.7%)	8 (13.8%)
BBs + Thiazides	2 (7.4%)	3 (1.8%)	3 (1.7%)	2 (1.3%)	1 (1.7%)
ACEIs + Thiazides	8 (29.6%)	28 (16.8%)	27 (15.5%)	6 (4.0%)	1 (1.7%)
ACEIs + BBs	3 (11.1%)	9 (5.3%)	7 (4.0%)	5 (3.3%)	2 (3.5%)
CCBs + ARBs	1 (3.7%)	18 (10.8%)	26 (14.9%)	37 (24.7%)	16 (27.6%)
ACEIs + Loop diuretics	–	1 (0.6%)	4 (2.3%)	5 (3.3%)	2 (3.5%)
BBs + ARBs	1 (3.7%)	7 (4.2%)	9 (5.2%)	7 (4.7%)	3 (5.2%)
BBs + CCBs	1 (3.7%)	7 (4.2%)	6 (3.5%)	11 (7.3%)	3 (5.2%)
BBs + Loop diuretics	–	2 (1.2%)	4 (2.3%)	4 (2.7%)	1 (1.7%)
Loop diuretics + ARBs	1 (3.7%)	5 (3.0%)	6 (3.5%)	8 (5.3%)	3 (5.2%)
Thiazides + CCBs	–	3 (1.8%)	8 (4.6%)	11 (7.3%)	6 (10.3%)
Loop diuretics + CCBs	–	1 (0.6%)	2 (1.2%)	10 (6.7%)	6 (10.3%)
Other dual therapy combinations	–	6 (3.6)	1 (0.6)	8 (5.3%)	3 (5.2%)
**Triple therapy*n* (%)**	**9** (**10.9%)**	**57** (**10.6%)**	**54** (**8.5%)**	**42** (**5.7%)**	**9** (**2.4%)**
**Quadruple therapy *n* (%)**	**3** (**3.6%)**	**12** (**2.2%)**	**12** (**1.9%)**	**7** (**0.9%)**	**3** (**0.8%)**

*BP data was collected up till 30th of March 2021.

**Missing readings.

^a^ SBP: Systolic BP.

^b^ DBP: Diastolic BP.

^c^ BBs: Beta-blockers; ACEIs: Angiotensin-converting enzyme inhibitors; CCBs: Calcium channel blockers; ARBs: Angiotensin receptor blockers; Vasodilators: Hydralazine; Imidazoline receptor agonists: Moxonidine; Multiple drug therapy refers to patients receiving more than 4 antihypertensives drug therapy.

#### Antihypertensives prescribing patters among patients with hypertension and Chronic Kidney Disease (CKD)

Fifteen patients with hypertension and CKD were treated with antihypertensives in 2017, including six patients (40.0%) on monotherapy. ARBs were the most commonly prescribed antihypertensives for patients on monotherapy (66.7%). The most common dual therapy combination was ACEIs + CCBs (50.0%), followed by ARBs + Thiazides (25.0%).

In 2018, 84 patients with CKD were treated for hypertension. There were 37 patients (44.0%) treated with monotherapy, and 26 patients (30.9%) treated with dual therapy. Among those on monotherapy, ARBs (35.1%) were the most frequently prescribed antihypertensives, followed by ACEIs (27.0%). Of those on dual therapy, the most prescribed combination was ARBs + Thiazides (34.6%), followed by ACEIs + CCBs (19.2%).

In 2019, 100 patients with hypertension and CKD received drug therapy, of which 56 (56%) were on monotherapy, and 31 (31%) were on dual therapy. ARBs (26.8%) and CCBs (25.0%) were the most regularly prescribed antihypertensives for patients on monotherapy. The most common combination among those receiving dual therapy was ARBs + Thiazides (32.2%), followed by CCBs + ARBs (16.1%).

Table 4 provides further details on antihypertensives patterns in hypertensive patients with CKD.

**Table 4.** Patterns of antihypertensives in patients with Chronic Kidney Disease (*N* = 218).

	Number of treated hypertensive patients				
	2017 *N* = 15	2018 *N* = 84	2019 *N* = 100	2020 *N* = 117	2021 *N* = 52
**Mean SBP**^a^**(SD)**	**	**137.6** (**15.4)**	**135.1** (**14.9)**	**137.0** (**15.7)**	**–**
**Mean DBP**^b^**(SD)**	**	**73.9** (**9.6)**	**72.1** (**9.2)**	**72.6** (**9.5)**	**–**
**Monotherapy**^a^***n* (%)**	**6 (40.0%)**	**37** (**44.0%)**	**56** (**56.0%)**	**64** (**54.7%)**	**36** (**69.2%)**
BB	–	1 (2.7%)	7 (12.5%)	5 (7.8%)	6 (16.7%)
ACEI	1 (16.7%)	10 (27.0%)	12 (21.4%)	5 (7.8%)	–
CCBs	1 (16.7%)	8 (21.6%)	14 (25.0%)	14 (21.9%)	16 (44.4%)
Loop diuretics	–	5 (13.5%)	7 (12.5%)	16 (25.0%)	6 (16.7%)
Thiazides	–	–	1 (1.8%)	2 (3.1%)	2 (5.6%)
ARBs	4 (66.7%)	13 (35.1%)	15 (26.8%)	19 (29.7%)	4 (11.1%)
Alpha2 agonists	–	–	–	1 (1.6%)	–
Direct vasodilators	–	–	–	1 (1.6%)	1 (2.8%)
**Dual Therapy**^c^ ***n* (%)**	**8 (53.3%)**	**26** (**30.9%)**	**31** (**31.0%)**	**37** (**31.6%)**	**12** (**23.1%)**
CCB + ACEI	4 (50.0%)	5 (19.2%)	3 (9.7%)	7 (18.9%)	–
ARBs + Thiazides	2 (25.0%)	9 (34.6%)	10 (32.3%)	1 (2.7%)	–
BBs + Thiazides	–	1 (3.9%)	1 (3.2%)	1 (2.7%)	–
CCBs + ARBs	–	3 (11.5%)	5 (16.1%)	8 (21.6%)	4 (33.3%)
ACEIs + ARBs	–	1 (3.9%)	1 (3.2%)	–	–
BBs + ARBs	1 (12.5%)	–	1 (3.2%)	1 (2.7%)	1 (8.3%)
BBs + CCBs	1 (12.5%)	1 (3.9%)	–	3 (8.1%)	1 (8.3%)
BBs + Loop diuretics	–	2 (7.7%)	3 (9.7%)	2 (5.4%)	–
Loop diuretics + ARBs	–	1 (3.9%)	2 (6.5%)	1 (2.7%)	–
Thiazides + CCBs	–	1 (3.9%)	1 (3.2%)	2 (5.4%)	1 (8.3%)
Loop diuretics + CCBs	–	1 (3.9%)	2 (6.5%)	5 (13.5%)	4 (33.3%)
Other dual therapy combinations	–	1 (3.9%)	2 (6.5%)	6 (16.2%)	1 (8.3%)
**Triple therapy *n* (%)**	**1 (6.7%)**	**17** (**20.2%)**	**9** (**9.0%)**	**14** (**11.9%)**	**3** (**5.7%)**
**Quadruple therapy** ***n* (%)**	**–**	**2** (**2.4%)**	**2** (**2.0%)**	**2** (**1.7%)**	**1** (**1.9%)**

*BP data was collected up till 30 March 2021.

**Missing readings.

^a^ SBP: Systolic BP

^b^ DBP: Diastolic BP

^c^ BBs: Beta-blockers; ACEIs: Angiotensin-converting enzyme inhibitors; CCBs: Calcium channel blockers; ARBs: Angiotensin receptor blockers; Vasodilators: Hydralazine; Imidazoline receptor agonists: Moxonidine; Multiple drug therapy refers to patients receiving more than 4 antihypertensives drug therapy.

#### Antihypertensives prescribing patterns among patients with hypertension and other comorbidities

Data on antihypertensives prescribing patterns in patients with hypertension and other comorbidities are presented in Table 5 (Congestive Heart Failure), Table 6 (Ischemic Heart Disease), and Table 7 (no known comorbidity).

**Table 5.** Patterns of antihypertensives in patients with Congestive Heart Failure (*N* = 113).

	Number of treated hypertensive patients				
	2017 *N* = 5	2018 *N* = 50	2019 *N* = 54	2020 *N* = 64	2021 *N* = 32
**Mean SBP**^a^**(SD)**	**	**133.4** (**14.3)**	**130.3** (**14.4)**	**129.5** (**15.8)**	**–**
**Mean DBP**^b^**(SD)**	**	**71.3** (**7.6)**	**69.0** (**8.4)**	**69.2** (**8.2)**	**–**
**Monotherapy**^c^***n* (%)**	**1 (20.0%)**	**22** (**44.0%)**	**30** (**55.6%)**	**29** (**45.3%)**	**18** (**56.2%)**
BB	1 (100.0%)	5 (22.7%)	3 (10.0%)	–	1 (5.6%)
ACEI	–	3 (13.6%)	4 (13.3%)	5 (17.2%)	4 (22.2%)
CCBs	–	4 (18.2%)	3 (10.0%)	4 (13.8%)	2 (11.1%)
Loop diuretics	–	8 (36.4%)	11 (36.7%)	15 (51.7%)	7 (38.9%)
Thiazides	–	–	–	1 (3.4%)	–
ARBs	–	2 (9.1%)	9 (30.0%)	3 (10.3%)	4 (22.2%)
Direct vasodilators	–	–	–	1 (3.4%)	–
**Dual Therapy**^c^***n* (%)**	**3 (60.0%)**	**17** (**34.0%)**	**15** (**27.8%)**	**26** (**40.6%)**	**11** (**34.3%)**
CCB + ACEI	–	1 (5.9%)	–	–	–
ARBs + Thiazides	–	5 (29.4%)	2 (13.3%)	2 (7.7%)	1 (9.1%)
ACEIs + BBs	1 (33.3%)	3 (17.7%)	2 (13.3%)	3 (11.5%)	–
CCBs + ARBs	–	1 (5.9%)	1 (6.7%)	3 (11.5%)	–
ACEIs + Loop diuretics	–	–	2 (13.3%)	3 (11.5%)	2 (18.2%)
BBs + Loop diuretics	–	2 (11.8%)	2 (13. 3%)	3 (11.5%)	1 (9.1%)
Loop diuretics + ARBs	1 (33.3%)	3 (17.7%)	3 (20.0%)	4 (15.4%)	3 (27.3%)
Loop diuretics + CCBs	–	1 (5.9%)	2 (13.3%)	5 (19.2%)	3 (27.3%)
Other dual therapy combinations	3 (100%)	1 (5.9%)	1 (6.7%)	3 (11.5%)	1 (9.1%)
**Triple therapy *n* (%)**	**1 (20.0%)**	**9** (**18.0%)**	**5** (**9.3%)**	**6** (**9.3%)**	**2** (**6.2%)**
**Quadruple therapy *n* (%)**	**–**	**2** (**4.0%)**	**3** (**5.6%)**	**3** (**4.7%)**	**1** (**3.1%)**

*BP data was collected up till 30 March 2021.

**Missing readings.

^a^ SBP: Systolic BP.

^b^ DBP: Diastolic BP.

^c^ BBs: Beta-blockers; ACEIs: Angiotensin-converting enzyme inhibitors; CCBs: Calcium channel blockers; ARBs: Angiotensin receptor blockers; Vasodilators: Hydralazine; Imidazoline receptor agonists: Moxonidine; Multiple drug therapy refers to patients receiving more than 4 antihypertensives drug therapy.

**Table 6.** Patterns of antihypertensives in patients with Ischemic Heart Disease (*N* = 106).

	Number of treated hypertensive patients				
	2017 *N* = 6	2018 *N* = 40	2019 *N* = 46	2020 *N* = 51	2021 *N* = 35
**Mean SBP**^a^**(SD)**	**	**136.9** (**15.5)**	**135.3** (**15.7)**	**135.5** (**16.6)**	**–**
**Mean DBP**^b^**(SD)**	**	**74.0** (**7.6)**	**74.1** (**8.9)**	**73.1** (**7.7)**	**–**
**Monotherapy**^c^***n* (%)**	**1 (16.7%)**	**21** (**52.5%)**	**24** (**52.2%)**	**28** (**54.9%)**	**26** (**74.3%)**
BB	–	1 (4.8%)	3 (12.5%)	2 (7.1%)	3 (11.5%)
ACEI	–	1 (4.8%)	3 (12.5%)	3 (10.7%)	2 (7.7%)
CCBs	–	5 (23.8%)	9 (37.5%)	6 (21.4%)	12 (46.2%)
Loop diuretics	–	6 (28.6%)	6 (25.0%)	9 (32.1%)	6 (23.1%)
Thiazides	–	3 (14.3%)	1 (4.2%)	–	1 (3.8%)
ARBs	1 (100.0%)	5 (23.8%)	2 (8.3%)	9 (32.1%)	2 (7.7%)
**Dual Therapy**^c^*n***(%)**	**4 (66.7%)**	**13** (**32.5%)**	**16** (**34.8%)**	**16** (**31.4%)**	**7** (**20.0%)**
CCB + ACEI	–	2 (15.4%)	–	–	1 (14.3%)
ARBs + Thiazides	1 (25.0%)	2 (15.4%)	6 (37.5%)	2 (12.5%)	1 (14.3%)
ACEIs + BBs	1 (25.0%)	1 (7.7%)	1 (6.3%)	1 (6.3%)	–
CCBs + ARBs	–	1 (7.7%)	3 (18.8%)	3 (18.8%)	2 (28.6%)
BBs + ARBs	1 (25.0%)	1 (7.7%)	–	–	1 (14.3%)
BBs + CCBs	1 (25.0%)	2 (15.4%)	1 (6.3%)	2 (12.5%)	–
BBs + Loop diuretics	–	2 (15.4%)	3 (18.8%)	3 (18.8%)	1 (14.3%)
Loop diuretics + ARBs	–	2 (15.4%)	1 (6.3%)	1 (6.3%)	
Other dual therapy combinations	–	–	1 (6.3%)	4 (25%)	1 (14.3%)
**Triple therapy *n* (%)**	**–**	**5** (**12.5%)**	**4** (**8.7%)**	**5** (**9.8%)**	**1** (**2.8%)**
**Quadruple therapy *n* (%)**	**1 (16.7%)**	**–**	**1** (**2.2%)**	**1** (**1.9%)**	**1** (**2.8%)**

*BP data was collected up till 30 March 2021.

**Missing readings.

^a^ SBP: Systolic BP.

^b^ DBP: Diastolic BP.

^c^ BBs: Beta-blockers; ACEIs: Angiotensin-converting enzyme inhibitors; CCBs: Calcium channel blockers; ARBs: Angiotensin receptor blockers; Vasodilators: Hydralazine; Imidazoline receptor agonists: Moxonidine; Multiple drug therapy refers to patients receiving more than 4 antihypertensives drug therapy.

**Table 7.** Patterns of anti-hypertensives in hypertensive patients with no comorbidities (*N* = 430).

	Number of treated hypertensive patients				
	2017 *N* = 6	2018 *N* = 108	2019 *N* = 206	2020 *N* = 255	2021 *N* = 113
**Mean SBP**^a^**(SD)**	**	**142.4** (**16.9)**	**139.9** (**16.1)**	**141.4** (**18.5)**	**–**
**Mean DBP**^b^**(SD)**	**	**85.3** (**9.1)**	**83.8** (**9.9)**	**84.4** (**11.6)**	**–**
**Monotherapy**^c^***n* (%)**	**5 (83.3%)**	**71** (**65.7%)**	**135** (**65.5%)**	**211** (**82.7%)**	**104** (**92.0%)**
BB	2 (40.0%)	10 (14.1%)	8 (5.9%)	7 (3.3%)	3 (2.9%)
ACEI	–	10 (14.1%)	28 (20.7%)	27 (12.8%)	15 (14.4%)
CCBs	2 (40.0%)	32 (45.1%)	61 (45.2%)	112 (53.1%)	56 (53.8%)
Loop diuretics	–	–	–	–	–
Thiazides	–	5 (7.0%)	7 (5.2%)	8 (3.8%)	7 (6.7%)
ARBs	1 (20.0%)	13 (18.3%)	28 (20.7%)	53 (25.1%)	20 (19.2%)
Alpha2 agonists	–	1 (1.4%)	3 (2.2%)	4 (1.9%)	3 (2.9%)
**Dual Therapy**^c^ ***n* (%)**	**1 (16.7%)**	**28** (**25.9%)**	**60** (**29.1%)**	**38** (**14.9%)**	**8** (**7.1%)**
CCB + ACEI	–	9 (32.1%)	23 (38.3%)	11 (28.9%)	2 (25.0%)
ARBs + Thiazides	1 (100.0%)	3 (10.7%)	10 (16.6%)	5 (13.2%)	–
BBs + Thiazides	–	2 (7.1%)	1 (1.7%)	–	1 (12.5%)
ACEIs + Thiazides	–	6 (21.4%)	14 (23.3%)	1 (2.6%)	–
ACEIs + BBs	–	–	1 (1.7%)	4 (10.5%)	–
CCBs + ARBs	–	1 (3.6%)	4 (6.7%)	8 (21.1%)	2 (25.0%)
BBs + ARBs	–	1 (3.6%)	–	2 (5.3%)	–
BBs + CCBs	–	1 (3.6%)	2 (3.3%)	–	–
Thiazides + CCBs	–	3 (10.7%)	2 (3.3%)	4 (10.5%)	3 (37.5%)
**Other dual therapy combinations**	**–**	2 (7.1%)	3 (5%)	3 (7.9%)	–
**Triple therapy*n* (%)**	**–**	**9** (**8.3%)**	**9** (**4.4%)**	**6** (**2.3%)**	**1** (**0.9%)**
**Quadruple therapy *n* (%)**	**–**	**–**	**1** (**0.5%)**	**–**	**–**

*BP data was collected up till 30 March 2021.

**Missing readings.

^a^ SBP: Systolic BP.

^b^ DBP: Diastolic BP.

^c^ BBs: Beta-blockers; ACEIs: Angiotensin-converting enzyme inhibitors; CCBs: Calcium channel blockers; ARBs: Angiotensin receptor blockers; Vasodilators: Hydralazine; Imidazoline receptor agonists: Moxonidine; Multiple drug therapy refers to patients receiving more than 4 antihypertensives drug therapy.

## Discussion

This study has contributed to a better understanding of the different prescribing patterns and trends of antihypertensives in the primary care setting in Qatar. The study results are considered a valuable addition to the limited published literature on this topic in Qatar. Overall, regardless of comorbidities or combination therapy, ACEIs were the most prescribed antihypertensives in 2017, but CCBs became the main antihypertensives from 2018 to 2021. The majority of the patients with hypertension received monotherapy across the years 2017, 2018, 2019, 2020, and 2021 regardless of their comorbidities. These results are comparable to other published studies, including a study conducted in India where monotherapy was prescribed more than combination therapy (Beg et al., 2014). Conversely, a study conducted to assess the prescribing patterns of antihypertensives in Nigeria, revealed that most patients with hypertension received polytherapy and their prescribing was in concordance with hypertension management guidelines (Adamu et al., 2017). Similar findings were observed in another study conducted in Nigeria (Adejumo et al., 2017).

The present study showed that among patients with diabetes treated with monotherapy, the most commonly prescribed antihypertensives were ACEIs and ARBs, which is in agreement with most published hypertension guidelines (James et al., 2014; National Institute for Health and Care Excellence, 2019; Whelton et al., 2018). For instance, the PHCC guidelines recommend the use of ACEIs or ARBs as first-line therapy for diabetic patients with hypertension (Primary Health Care Corporation, 2020; National Institute for Health and Care Excellence, 2019). While the ACC/AHA guidelines recommend the use of any of the first line agents, including ACEIs, ARBs, and CCBs, for patients with diabetes and hypertension (Whelton et al., 2018). Moreover, the evidence demonstrates the importance of using ACEIs/ARBs in patients with hypertension and diabetes. One meta-analysis of randomised controlled trials proved the effectiveness of ACEIs/ARBs in reducing cardiovascular events and mortality rates in hypertensive patients with diabetes.(Hao et al., 2014) The most common combinations of antihypertensives prescribed to diabetic patients were ACEIs/ARBs + Thiazides, and ACEIs/ARBs + CCBs, which is also in accordance with the latest clinical practice guidelines’ recommendations (James et al., 2014; National Institute for Health and Care Excellence, 2019; Whelton et al., 2018). In fact, the PHCC guidelines recommend using the combination of ACEIs/ARBs + CCBs/Thiazide-like diuretics for hypertensive patients with type 2 diabetes mellitus (Primary Health Care Corporation, 2020; National Institute for Health and Care Excellence, 2019). While the ACC/AHA guidelines recommend their renoprotective effects in diabetic patients (Whelton et al., 2018). Furthermore, the study findings are comparable to others identifying ACEIs/ARBs and CCBs as the most frequently prescribed combination among hypertensive patients with diabetes (Abougalambou et al., 2011; Ibaraki et al., 2017; Ishida et al., 2019; Rajasekhar et al., 2016; Yazdanshenas et al., 2014).

Furthermore, among patients treated with monotherapy for CKD, ARBs, ACEIs, and CCBs were the most commonly prescribed antihypertensives, which is equally in line with guidelines recommendations (James et al., 2014; Levin & Stevens, 2014; National Institute for Health and Care Excellence, 2019; Whelton et al., 2018). Among those receiving combination antihypertensive therapy, the most prescribed combinations were ACEIs/ARBs + CCBs, and ARBs + Thiazides, which is consistent with the latest guidelines recommendations for hypertension management in CKD patients (James et al., 2014; Levin & Stevens, 2014; National Institute for Health and Care Excellence, 2019; Whelton et al., 2018). The ACC/AHA guidelines commend using ACEIs to slow kidney disease progression and suggest using ARBs in case of intolerance to ACEIs (Whelton et al., 2018). The use of ACEIs, ARBs, and CCBs is supported by several studies in the literature due to their renal protective properties, including reducing kidney failure risk in addition to their ability to decrease cardiovascular-related morbidity and mortality (Lin et al., 2017; Xie et al., 2016).

Monotherapy antihypertensives prescribed to Ischemic Heart Disease (IHD) patients included ARBs, CCBs, and loop diuretics, which is not in accordance with guidelines for hypertension management in CVD patients, since BBs should be prescribed unless contraindicated (Whelton et al., 2018). The ACC/AHA guidelines recommend treating adults with stable IHD with BBs, ACEIs, or ARBs first and adding dihydropyridine CCBs, thiazide diuretics, and/or mineralocorticoid receptor antagonists as needed to reach target blood pressure (Whelton et al., 2018). This observed lack of concordance with guidelines may be attributed to the PHCC guidelines which do not recommend treatment for patients with IHD or CVD. As a result, physicians following only the PHCC guidelines are less likely to prescribe BBs.

Furthermore, this study established that among patients with CHF, the most prescribed monotherapy agents were BBs, loop diuretics, and ARBs which is in agreement with guidelines recommendations (Whelton et al., 2018), and with other studies that assessed the prescribing patterns of antihypertensives among patients with Congestive Heart Failure (CHF) (Kim et al., 2019). The ACC/AHA guidelines recommend the use of ACEIs or ARBs, and BBs in adult patients with CHF with preserved ejection fraction and with volume overload (Whelton et al., 2018). The use of ARBs in managing hypertensive patients with CHF was also investigated in a review of the literature and ARBs were proven effective as they provide a protective effect against heart failure, stroke, and proteinuria risks (Omboni & Volpe, 2018).

BBs and CCBs were the most prescribed monotherapy agents among hypertensive patients without other known comorbidities in 2017, which is not in line with hypertension guidelines, as BBs are not recommended as first-line therapy for newly diagnosed hypertensive patients (Primary Health Care Corporation 2016; James et al., 2014; Whelton et al., 2018). There might be reasons for this lack of compliance with national and international hypertension management guidelines, for example physicians not following the latest guidelines or the lack of documentation of comorbidities in the Cerner® system when it was established in 2017. Moreover, detailed information regarding patients’ allergies, contraindications, and other comorbidities was not provided; thus, this may have affected the interpretation of the apparent incongruence with guidelines. It is important to highlight that only six hypertensive patients were identified without any known comorbidities in 2017, which may impact the generalisability of these findings. This limited sample size is primarily due to the fact that most hypertensive patients have additional comorbidities, leaving only a small proportion with hypertension as their only medical condition.

However, from 2018 to 2021 the most prescribed monotherapy agents were CCBs, ARBs, and ACEIs which is in concordance with hypertension guidelines’ recommendations in hypertensive patients without known comorbidities (James et al., 2014; Whelton et al., 2018). Comparatively, according to a 2019 study conducted in the Kingdom of Saudi Arabia (KSA) Al Aseer region, antihypertensive prescribing followed the JNC8 hypertension management guidelines as well as KSA regional hypertension guidelines (Siddiqua et al., 2019). The authors still felt further improvements were needed in prescribing antihypertensives, which is consistent with our study findings (Siddiqua et al., 2019).

As for the antihypertensives trends, there has been an increase in the prescribing of CCBs over the last five years, as well as a decrease in the prescribing of thiazides and ACEIs. According to a study published in 2019, prescribing trends for thiazide diuretics in the United Kingdom have decreased over time (McNally et al., 2019). The authors attributed the decline in thiazide diuretic prescriptions to the 2011 NICE hypertension management guidelines that recommended first-line therapy with ACEIs/ARBs or CCBs (McNally et al., 2019). Moreover, they suggested that other factors could have contributed such as patients’ non-adherence to therapy due to thiazide diuretics’ side effects including frequent urination, fatigue, and electrolyte disturbances which may have led to discontinuation of therapy (McNally et al., 2019). In 2013, another study of prescribing trends in Hong Kong concluded that ACEI prescribing decreased while CCB prescribing remained stable (Wong et al., 2013).

Overall, the study findings established that the prescribing patterns and trends of antihypertensives in the primary care setting in Qatar are mostly in accordance with national and international hypertension guidelines recommendations except for a few prescriptions. These findings are highly rewarding for Qatar and confirm the compliance of Qatar’s healthcare system with international standards. Moreover, the study results demonstrate that Qatar strives to offer the best quality health care to its citizens and residents. Additionally, it is obvious that Qatar is working towards achieving Qatar Vision 2030 goals and Qatar Primary Care strategy of improving the health of its people and decreasing the burden of NCDs (Qatar National Vision, 2030, 2008; Qatar Primary Care Strategy, 2019). However, there is always room for improvement in hypertension management in Qatar; thus, further strategies for improvement are always an exciting area to investigate in future research. Examples of these strategies include conducting regular continuing professional education workshops on hypertension management for PHCC physicians.

### Limitations

There were, however, some challenges encountered in this study that resulted in a few limitations, including the researchers’ inability to personally extract all patients’ data and records from Cerner®, limiting the amount of data that could be extracted. For example, researchers were unable to retrieve patients’ family history, body mass index, lifestyle, or smoking habits. In order to overcome this limitation, regular meetings were held with the IT team to ensure the extraction of as much relevant data as possible. Therefore, physicians’ adherence to guidelines and comparison of the prescribing patterns of antihypertensives to guidelines’ recommendations was not intended as a study goal due to the missing data above. Furthermore, Cerner ® records were not created until 2017; therefore, extracting records before this data was not possible.

### Recommendations for future studies

In order to prevent hypertension-associated complications, physicians must prescribe antihypertensives and manage hypertension according to the latest hypertension management guidelines. Therefore, further research is recommended to determine physicians’ adherence to clinical guidelines when treating hypertension using prospective data, with adherence to guidelines being the most important outcome. Furthermore, physicians must attend Continuous Professional Development (CPD) workshops to stay current with the latest guidelines for the management of hypertension. Additionally, further research should be conducted to determine the impact of these workshops on hypertension management in primary care settings.

## Conclusion

The findings of this study offer valuable insights that can aid clinicians and policymakers in optimising the use of antihypertensive agents. By identifying trends and gaps in current practice, these insights pave the way for better-targeted interventions, which in turn can lead to improved patient outcomes and more efficient utilisation of healthcare resources. This is especially critical for guiding both policy and treatment approaches in line with updated clinical guidelines. Moreover, understanding these patterns helps highlight potential discrepancies in care and areas for further improvement, ensuring that clinical guidelines are effectively translated into everyday practice.

## Data Availability

Data is available on a reasonable request.

## References

[CIT0009] AbdulRashid, R., Hameed, R., & Hameed, T. (2021). Prevalence of hypertension among the adult population in Qatar: A literature review. *Scholars Journal of Applied Medical Sciences*, *9*(12), 1805–1812. 10.36347/sjams.2021.v09i12.008

[CIT0010] Abougalambou, S. S., Abougalambou, A. S., Sulaiman, S. A., & Hassali, M. A. (2011). Prevalence of hypertension, control of blood pressure and treatment in hypertensive with type 2 diabetes in Hospital University Sains Malaysia. *Diabetes & Metabolic Syndrome*, *5*(3), 115–119. 10.1016/j.dsx.2012.03.001 22813562

[CIT0011] Adamu, U., Abdulahi, A., Ibrahim, F., & Ibok, I. (2017). Pattern of medication use among hypertensives attending a specialist outpatients clinic in North-Central Nigeria. *Journal of Pharmaceutical Research International*, *17*(3), 1–8.

[CIT0012] Adejumo, O., Okaka, E., & Iyawe, I. (2017). Prescription pattern of antihypertensive medications and blood pressure control among hypertensive outpatients at the University of Benin Teaching Hospital in Benin City, Nigeria. *Malawi Medical Journal*, *29*(2), 113–117. 10.4314/mmj.v29i2.7 28955417 PMC5610280

[CIT0013] Al Thani, A., Fthenou, E., Paparrodopoulos, S., Al Marri, A., Shi, Z., Qafoud, F., & Afifi, N. (2019). Qatar biobank cohort study: Study design and first results. *American Journal of Epidemiology*, *188*(8), 1420–1433. 10.1093/aje/kwz084 30927351

[CIT0014] Ambrose, J. A., & Barua, R. S. (2004). The pathophysiology of cigarette smoking and cardiovascular disease: An update. *Journal of the American College of Cardiology*, *43*(10), 1731–1737. 10.1016/j.jacc.2003.12.047 15145091

[CIT0015] Beg, M., Dutta, S., Varma, A., Kant, R., Bawa, S., Anjoom, M., Sindhu, S., & Kumar, S. (2014). Study on drug prescribing pattern in hypertensive patients in a tertiary care teaching hospital at Dehradun, Uttarakhand. *International Journal of Medical Science and Public Health*, *3*(8), 922.

[CIT0016] Canoy, D., Copland, E., Nazarzadeh, M., Ramakrishnan, R., Pinho-Gomes, A. C., Salam, A., Dwyer, J. P., Farzadfar, F., Sundstrom, J., Woodward, M., Davis, B. R., & Rahimi, K. (2022). Antihypertensive drug effects on long-term blood pressure: An individual-level data meta-analysis of randomised clinical trials. *Heart*, *108*(16), 1281–1289. 10.1136/heartjnl-2021-320171 35058294 PMC9340038

[CIT0017] Chobanian, A. V., Bakris, G. L., Black, H. R., Cushman, W. C., Green, L. A., Izzo, J. L., Jones, D. W., Materson, B. J., Oparil, S., Wright, J. T., & Roccella, E. J. (2003). The Seventh Report of the Joint National Committee on prevention, detection, evaluation, and treatment of high blood pressure: The JNC 7 report. *JAMA*, *289*(19), 2560–2572. 10.1001/jama.289.19.2560 12748199

[CIT0018] *Clinical guidelines for the State of Qatar*. (2019). https://www.moph.gov.qa/Admin/Lists/ClinicalGuidelinesAttachments/Attachments/23/The%20diagnosis%20and%20management%20of%20hypertension%20in%20adults.pdf

[CIT0019] Cuspidi, C., Tadic, M., Grassi, G., & Mancia, G. (2018). Treatment of hypertension: The ESH/ESC guidelines recommendations. *Pharmacological Research*, *128*, 315–321. 10.1016/j.phrs.2017.10.003 29080798

[CIT0020] Dorans, K. S., Mills, K. T., Liu, Y., & He, J. (2018). Trends in prevalence and control of hypertension according to the 2017 American College of Cardiology/American Heart Association (ACC/AHA) Guideline. *Journal of the American Heart Association*, *7*(11), e008888. 10.1161/JAHA.118.008888 PMC6015372 29858369

[CIT0021] Ettehad, D., Emdin, C. A., Kiran, A., Anderson, S. G., Callender, T., Emberson, J., Chalmers, J., Rodgers, A., & Rahimi, K. (2016). Blood pressure lowering for prevention of cardiovascular disease and death: A systematic review and meta-analysis. *Lancet*, *387*(10022), 957–967. 10.1016/S0140-6736(15)01225-8 26724178

[CIT0022] Hao, G., Wang, Z., Guo, R., Chen, Z., Wang, X., Zhang, L., & Li, W. (2014). Effects of ACEI/ARB in hypertensive patients with type 2 diabetes mellitus: A meta-analysis of randomized controlled studies. *BMC Cardiovascular Disorders*, *14*, 148. 10.1186/1471-2261-14-148 25344747 PMC4221690

[CIT1001] Hamad Medical Corporation. (2023). *Health Care in Qatar*. https://www.hamad.qa/EN/Quick%20Links/Health%20Care%20in%20Qatar/Pages/default.aspx

[CIT1002] *Hypertension key facts*. (2021). Retrieved August 28, 2022, from https://www.who.int/news-room/fact-sheets/detail/hypertension

[CIT0023] Ibaraki, A., Goto, W., Iura, R., Tominaga, M., & Tsuchihashi, T. (2017). Current prescription status of antihypertensive drugs with special reference to the use of diuretics in Japan. *Hypertension Research*, *40*(2), 203–206. 10.1038/hr.2016.120 27581534

[CIT1003] *ICD-11*. (n.d.). https://icd.who.int/dev11/f/en#/http%3a%2f%2fid.who.int%2ficd%2fentity%2f1434352150

[CIT0024] Ishida, T., Oh, A., Hiroi, S., Shimasaki, Y., & Tsuchihashi, T. (2019). Current prescription status of antihypertensive drugs in Japanese patients with hypertension: Analysis by type of comorbidities. *Clinical and Experimental Hypertension*, *41*(3), 203–210. 10.1080/10641963.2018.1465074 29781721

[CIT0025] James, P. A., Oparil, S., Carter, B. L., Cushman, W. C., Dennison-Himmelfarb, C., Handler, J., Lackland, D. T., LeFevre, M. L., MacKenzie, T. D., Ogedegbe, O., Smith, S. C., Svetkey, L. P., Taler, S. J., Townsend, R. R., Wright, J. T., Narva, A. S., & Ortiz, E. (2014). 2014 evidence-based guideline for the management of high blood pressure in adults: Report from the panel members appointed to the Eighth Joint National Committee (JNC 8). *JAMA*, *311*(5), 507–520. 10.1001/jama.2013.284427 24352797

[CIT0026] Kim, S. H., Shin, D. W., Kim, S., Han, K., Park, S. H., Kim, Y. H., Jeon, S. A., & Kwon, Y. C. (2019). Prescribing patterns of antihypertensives for treatment-naïve patients in South Korea: From Korean NHISS claim data. *International Journal of Hypertension*, *2019*, 4735876. 10.1155/2019/4735876 31534797 PMC6732595

[CIT0027] Levin, A., & Stevens, P. E. (2014). Summary of KDIGO 2012 CKD guideline: Behind the scenes, need for guidance, and a framework for moving forward. *Kidney International*, *85*(1), 49–61. 10.1038/ki.2013.444 24284513

[CIT0028] Lin, Y.-C., Lin, J.-W., Wu, M.-S., Chen, K.-C., Peng, C.-C., & Kang, Y.-N. (2017). Effects of calcium channel blockers comparing to angiotensin-converting enzyme inhibitors and angiotensin receptor blockers in patients with hypertension and chronic kidney disease stage 3 to 5 and dialysis: A systematic review and meta-analysis. *PLoS One*, *12*(12), e0188975. 10.1371/journal.pone.0188975 29240784 PMC5730188

[CIT0029] Maclaughlin, E. J. *Pharmacotherapy: A pathophysiologic approach, 11e NY*. McGraw-Hill Education.

[CIT0030] McNally, R. J., Morselli, F., Farukh, B., Chowienczyk, P. J., & Faconti, L. (2019). A review of the prescribing trend of thiazide-type and thiazide-like diuretics in hypertension: A UK perspective. *British Journal of Clinical Pharmacology*, *85*(12), 2707–2713. 10.1111/bcp.14109 31471972 PMC6955404

[CIT1004] National Institute for Health and Care Excellence. (2019). *Hypertension in adults: Diagnosis and management NG136*. https://www.niceorg.uk/guidance/ng136 31577399

[CIT0031] Omboni, S., & Volpe, M. (2018). Management of arterial hypertension with angiotensin receptor blockers: Current evidence and the role of olmesartan. *Cardiovascular Therapeutics*, *36*(6), e12471. 10.1111/1755-5922.12471 30358114 PMC6587798

[CIT1006] Primary Health Care Corporation. (n.d.). *Primary health care centers*. https://www.phcc.gov.qa/Health-Centers/All-Health-Centers

[CIT1007] Primary Health Care Corporation. (2016). *Clinical practice guideline for diagnosis and management of hypertension in adults in Qatar.* https://www.phcc.gov.qa

[CIT1008] Primary Health Care Corporation. (2020). *Clinical practice guideline for diagnosis and management of hypertension in adults in Qatar.* https://www.phcc.gov.qa

[CIT0032] *Qatar National Vision 2030*. (2008). https://www.psa.gov.qa/en/qnv1/Documents/QNV2030_English_v2.pdf

[CIT0033] *Qatar Primary Care Strategy*. (2019). https://www.moph.gov.qa/english/strategies/Supporting-Strategies-and-Frameworks/PrimaryHealthCareFoundationStrategy/Pages/default.aspx

[CIT0034] Rajasekhar, D. G., Prasanna, D. G., & Chandrakanth, P. (2016). Prescribing pattern of antihypertensive drugs based on compelling indications with hypertension. *International Journal of Pharmacy and Pharmaceutical Sciences*, *8*(2), 72–75. https://innovareacademics.in/journals/index.php/ijpps/article/view/6970

[CIT0035] Siddiqua, A., Alshehri, A., Alahmari, A. M., Alshehri, R. A., & Badawy, S. S. (2019). A study of prescription pattern and compliance of anti-hypertensives with the treatment guidelines in Aseer Region; Saudi Arabia. *Current Drug Targets*, *14*(3), 261–266.

[CIT0036] Whelton, P. K., Carey, R. M., Aronow, W. S., Casey, D. E., Collins, K. J., Dennison Himmelfarb, C., DePalma, S. M., Gidding, S., Jamerson, K. A., Jones, D. W., MacLaughlin, E. J., Muntner, P., Ovbiagele, B., Smith, S. C., Spencer, C. C., Stafford, R. S., Taler, S. J., Thomas, R. J., Williams, K. A., … Wright, J. T. (2018). 2017 ACC/AHA/AAPA/ABC/ACPM/AGS/APhA/ASH/ASPC/NMA/PCNA guideline for the prevention, detection, evaluation, and management of high blood pressure in adults: A report of the American College of Cardiology/American Heart Association Task Force on Clinical Practice Guidelines. *Journal of the American College of Cardiology*, *71*(19), e127–e248. 10.1016/j.jacc.2017.11.006 29146535

[CIT0037] Wong, M. C. S., Tam, W. W. S., Cheung, C. S. K., Tong, E. L. H., Sek, A. C. H., Cheung, N. T., Yan, B. P. Y., Yu, C.-M., & Griffiths, S. M. (2013). Antihypertensive prescriptions over a 10-year period in a large Chinese population. *American Journal of Hypertension*, *26*(7), 931–938. 10.1093/ajh/hpt049 23591987

[CIT0008] World Health Organization. (2018). *Noncommunicable diseases country profiles.* https://www.who.int/nmh/countries/e

[CIT0038] Xie, X., Liu, Y., Perkovic, V., Li, X., Ninomiya, T., Hou, W., Zhao, N., Liu, L., Lv, J., Zhang, H., & Wang, H. (2016). Renin-angiotensin system inhibitors and kidney and cardiovascular outcomes in patients with CKD: A Bayesian network meta-analysis of randomized clinical trials. *American Journal of Kidney Diseases*, *67*(5), 728–741. 10.1053/j.ajkd.2015.10.011 26597926

[CIT0039] Yazdanshenas, H., Bazargan, M., Orum, G., Loni, L., Mahabadi, N., & Husaini, B. (2014). Prescribing patterns in the treatment of hypertension among underserved African American elderly. *Ethnicity & Disease*, *24*(4), 431–437. 25417425 PMC4286375

